# Association between lifestyle and dietary preference factors and conventional adenomas and serrated polyps

**DOI:** 10.3389/fnut.2023.1269629

**Published:** 2024-01-10

**Authors:** Jue Xu, Peihan Chi, Kang Qin, Biao Li, Zhongxue Cheng, Zhecong Yu, Caixia Jiang, Yunxian Yu

**Affiliations:** ^1^HangZhou Center for Disease Control and Prevention, Hangzhou, China; ^2^Department of Epidemiology and Health Statistics, School of Public Health, School of Medicine, Zhejiang University, Hangzhou, China

**Keywords:** colorectal cancer, conventional adenomas, serrated polyps, modifiable lifestyle factors, diet

## Abstract

**Introduction:**

Both conventional adenoma (AD) and serrated polyp (SP) were known precursor lesions of colorectal cancer (CRC). Modifiable lifestyle factors were significantly associated with CRC risk, but whether these factors were related to the risk of different precursors of CRC needed to be clarified. This study aimed to evaluate the risks of AD and SP caused by lifestyle factors and compare the risk differences between AD and SP.

**Methods:**

The study population was from the CRC screening cohort in Hangzhou, China. A total of 458,457 eligible individuals volunteered to undergo initial screening including the fecal immunochemical test (FIT) and the CRC risk assessment. Finally, 13,993 participants who had undergone colonoscopy tests and had been diagnosed at designated hospitals were selected in this study. All participants were required to fill out a questionnaire during the initial screening for collecting their information. The generalized estimate equation (GEE) model was used to assess the association between lifestyle factors/dietary preferences and AD/SP.

**Results:**

The body mass index (BMI) and smoking were positively associated with the risks of only SP (BMI: OR = 1.50, 95%CI: 1.23–1.84; smoking: OR = 1.29, 95%CI: 1.07–1.55), only AD (BMI: OR = 1.53, 95%CI: 1.28–1.82; OR = 1.24, 95%CI: 1.11–1.39), and synchronous SP and AD (BMI: OR = 1.97, 95%CI: 1.40–2.75; smoking: OR = 1.53, 95%CI: 1.27–1.85). In the case-group comparison, smoking was more strongly associated with the risk of synchronous SP and AD than only AD. Alcohol drinking was positively associated with the risk of AD (OR = 1.28, 95%CI: 1.14–1.44), but no statistically significant difference was observed in risks in the case-group comparison. Furthermore, whole-grain intake was associated with a decreased risk of only AD (OR = 0.78, 95%CI: 0.65–0.93). However, white meat intake was positively associated with risks of only SP when compared with AD cases (OR = 1.60, 95%CI: 1.15–2.23).

**Conclusion:**

The current study identified common risk factors such as BMI and smoking as well as different risks of certain factors (e.g., alcohol drinking and whole-grain intake) for SP and AD. However, there were still some factors, especially diet-related factors, that have not been fully elucidated in their association with the two lesions. Further research is needed in future to confirm and develop prevention strategies for different lesions.

## 1 Introduction

With the development of the social economy, the transformation of population structure and the disease spectrum, cancer has become one of the leading causes of premature death (<70 years old) in most countries (1). Among them, colorectal cancer (CRC) ranks as the third most common malignant tumor worldwide. New cases and deaths of CRC were estimated to account for one-tenth of all cancer cases in 2020 (2). For decades, it was generally believed that conventional adenoma (AD) was the only known precursor lesion of CRC (3), caused by abnormal cell proliferation or DNA mismatch repair (4), and ultimately formed invasive cancer via the adenoma–carcinoma sequence. However, along with the improvement of detection technology, it has been found that CRC could be derived from another precursor pathway (5). Through the serrated-neoplasia pathway, serrated polyp (SP) has been estimated to give rise to approximately 15–30% of all CRC cases (3). According to the World Health Organization (WHO) classification of tumors of the digestive tract, SP has three known subtypes: hyperplastic polyp (HP), sessile serrated lesion (SSL), and traditional serrated adenomas (TSA) (6). These precursors, particularly the SSL subtype, were more frequently found in post-colonoscopy CRC, namely interval colorectal cancer (7), leading to the speculation that there was a high miss-rate of SPs in endoscopy or the serrated-neoplasia pathway would accelerate carcinogenesis (8). Therefore, serrated lesions with malignant potential have attracted increasing research attention; meanwhile, CRC was regarded as a heterogeneous disease.

Modifiable lifestyle factors were significantly associated with colorectal cancer (9). Smoking, alcohol intake, red meat, and processed meat consumption have been revealed to be risk factors for causing CRC in humans (10, 11). Thus, healthy changes in principal lifestyle factors can effectively prevent new CRC (12). However, do lifestyle risk factors show heterogeneity in the adenoma–carcinoma and serrated-neoplasia pathways? A few studies (13–16) have assessed whether these factors differed between different lesions and indicated the etiologic heterogeneity between the two precursor pathways. However, the current epidemiological evidence is inconsistent. For example, the association between alcohol intake and the serrated pathway has shown opposite results across the literature (14, 15). In addition, most of the evidence was limited by the sample size (15). Thus, large sample sizes of data were still needed to confirm the results of previous studies. In order to explore the role of lifestyle factors in different pathways of CRC, this study was based on a large population screening cohort in eastern China and aimed to evaluate the risks of conventional adenoma (AD) and serrated polyp (SP) caused by modifiable lifestyle factors and compare the risk differences between AD and SP.

## 2 Methods

### 2.1 Study population

**Figure 1:**
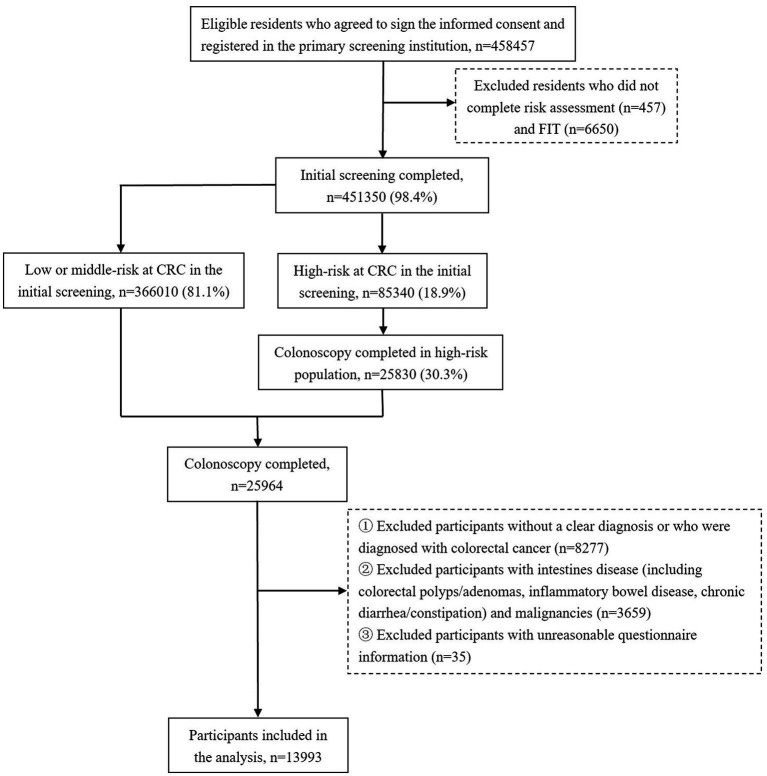
Flow chart of participants inclusion in the analysis.

This study was implemented in Hangzhou City, Zhejiang Province, China, from April 2020 to October 2020, for which residents aged 40 to 74 years were invited for a two-step CRC screening. The fecal immunochemical test (FIT) combined with the CRC risk assessment was adopted as the initial screening method, and colonoscopy was for the diagnostic examination. People assessed as high risk in FIT or risk assessment were both invited to undergo further testing. The high-risk threshold set for FIT was 20ug/g (100 ng/mL). The CRC risk assessment criteria (Supplementary material 1) were optimized on the basis of the Asia-Pacific Colorectal Screening scoring (17). In the end, a total of 458,457 eligible individuals volunteered to participate in the initial screening, and among them, 85,340 participants were assessed as being high risk for CRC, who were invited to take a colonoscopy examination for further diagnosis. During the screening period, 30.3% of the high-risk participants underwent a colonoscopy. In addition, a small number of residents at middle or low risk for CRC took the initiative to participate in the diagnostic screening. Therefore, a total of 25,964 participants underwent a colonoscopy examination. After excluding those with no colonoscopy diagnostic result, or with a diagnosis of non-adenomatous polyps or CRC, or with unreasonable questionnaire information, or with a previous diagnosis of intestines disease, including inflammatory bowel disease (IBD), colorectal adenomas, polyps, chronic diarrhea or constipation, and with previous malignancies, a total of 13,993 individuals were recruited in this study. Overall, the number of only SP cases, only AD cases, and synchronous SP and AD cases were 4,233, 1,097, and 638, respectively. The flow chart of the study sample is shown in Figure 1.

### 2.2 Variable definition

In the initial screening, participants were required to fill out a questionnaire through a face-to-face survey for their detailed information, which was reported by the participants themselves. The questionnaire was based on the questionnaire of the Cancer Screening Program in Urban China (CanSPUC), which was a national major public health service project in China (18). Data collected from the participants included sociodemographic characteristics and lifestyle factors relevant to CRC, such as sex, age, body weight and height, education status, marital status, history of diagnosed diseases including malignant tumors and intestine diseases, first-degree relative’s family history of CRC and familial adenomatous polyps, regular aspirin use, cigarette smoking, alcohol drinking, physical activity, and dietary preference. Body mass index (BMI) was calculated as weight (kg) divided by height (m) squared and was classified into low weight (<18.5 kg/m^2^), normal weight (18.5–23.9 kg/m^2^), overweight (24–27.9 kg/m^2^), and obesity (≥28 kg/m^2^) according to the guideline for the prevention and control of overweight and obesity in Chinese adults (19). Regular non-steroidal anti-inflammatory drugs (NSAIDs) use was defined as taking NSAIDs more than once a week. Smoking was categorized into never smoking, current smoking (more than 1 cigarette per day for more than 6 months), and former smoking (stop smoking for 2 years). Drinking was categorized into never drinking, current drinking (more than 1 time per week for more than 6 months), and former drinking (stop drinking for 2 years). The questionnaire asked residents how often they had participated in physical activity over the past year and divided it into five categories: never, 1–3 times per month, 1–2 times per week, 3–5 times per week, and every day or almost every day. The dietary preference survey asked participants how often they ate a variety of foods over the past year, with optional frequencies, including never, 1–3 days per month, 1–3 days per week, 4–6 days per week, and every day or almost every day. The food was categorized into types of diet, including vegetables, fruits, red meat, white meat, beans and soy products, preserved vegetables, processed meats, fried or grilled food, and whole grains.

All histological diagnosis was made by experienced physicians at designated hospitals, and detailed endoscopic data were recorded following a standard template, including bowel preparation, procedure completion, lesion location, type, size, morphology, and so on. The SP cases referred to individuals with HPs, SSLs, and TSAs. The AD cases referred to individuals with tubular adenomas, villous adenomas, tubular villous adenomas, and adenomas with dysplasia. If a participant had both SP and AD characteristics in the lesions at the same site, or a participant had both SP and AD lesions at a different site, then we regarded him as a synchronous SP and AD case in the current study.

### 2.3 Statistical analysis

Categorical variables were presented as frequency and percentage. The chi-square test was used to compare differences between groups of no polyp control group (including individuals with normal colonoscopy results or chronic colorectal inflammation), only SP cases, only AD cases, and synchronous SP and AD cases. Due to the participants from different districts, the relevant characteristics of participants were different across districts; therefore, the multivariate generalized estimate equation (GEE) model was used to assess the risk of lifestyle factors and dietary preferences for SP and AD, and the trend test was carried out for factors in ascending or descending categories. Sex, age group, education status, family history of CRC or familial adenomatous polyposis in first-degree relatives, cigarette smoking status, alcohol drinking status, frequency of physical activity, regular NSAID use, and dietary preference were adjusted in the model. In addition, the associations of a number of only SP and only AD cases with influencing factors were analyzed, respectively. In the analysis, duration, intensity, and quitting time of cigarette smoking and alcohol drinking were treated in tertile variables, respectively. R software (version 4.0.3) and SAS Studio were used for statistical analysis, and SAS Studio was mainly used for GEE model analysis. The value of *p* was considered as significant when less than 0.05 in a two-sided test. However, due to the multiple comparisons, the statistically significant level was adjusted by the false discovery rate (FDR).

## 3 Results

Table 1 presents demographic characteristics of the no polyp control group, only SP group, only AD group, and synchronous SP and AD groups. The detection rates of only SP, only AD, and synchronous SP and AD were higher in men than those in women (*p* < 0.001). Individuals aged 70–74 years showed the highest detection rate of only AD (40.2%) and synchronous SP and AD (5.6%), while those who aged 50–59 and 60–69 years had higher detection rates of only SP (8.3 and 8.1%) than the 70–74-year group (6.8%) (*p* < 0.001). Furthermore, significant differences between the four groups were also observed in the comparison of education status (*p* = 0.017) and family history of CRC (*p* < 0.001). There was a higher detection rate of only AD in the group who were illiterate (31.4%), but the detection rate of only SP was higher in those with a college education or above (9.3%). In addition, the detection rate of synchronous SP and AD was highest in the group with high school education or below. Participants who had a first-degree relative’s family history of CRC showed the highest prevalence of only AD (39.8%) and synchronous SP and AD (8.0%), but the prevalence of only SP was the lowest (6.6%). Individuals who had no idea about whether they had a family history of CRC exhibited the highest detection rate of only SP (9.6%). In addition, there were statistically significant differences in smoking and drinking status, physical activity, and regular aspirin use between the four groups.

**Table 1:** Distribution of sociodemographic characteristics between no polyp controls, only SP cases, only AD cases, and synchronous SP and AD groups.

Characteristic	Total (*n* = 13,993)	No polyp control (*n* = 8,025)		Only serrated polyp (SP) (*n* = 1,097)		Only conventional adenoma (AD) (*n* = 4,233)		Synchronous SP and AD (*n* = 638)		*p*
	*N* (%)	*N* (%)	Detection rate (%)	*N*	Detection rate (%)	*N*	Detection rate (%)	*N*	Detection rate (%)	
Sex										**<0.001**
Male	7,691 (55.0)	3,710 (46.2)	48.2	671 (61.2)	8.7	2,859 (67.5)	37.2	451 (70.7)	5.9	
Female	6,302 (45.0)	4,315 (53.8)	68.5	426 (38.8)	6.8	1,374 (32.5)	21.8	187 (29.3)	3.0	
Age group, years										**<0.001**
40~	248 (1.8)	185 (2.3)	74.6	12 (1.1)	4.8	43 (1.0)	17.3	8 (1.3)	3.2	
50~	4,527 (32.4)	3,071 (38.3)	67.8	375 (34.2)	8.3	957 (22.6)	21.1	124 (19.4)	2.7	
60~	6,469 (46.2)	3,466 (43.2)	53.6	522 (47.6)	8.1	2,128 (50.3)	32.9	353 (55.3)	5.5	
70 ~ 74	2,749 (19.6)	1,303 (16.2)	47.4	188 (17.1)	6.8	1,105 (26.1)	40.2	153 (24.0)	5.6	
BMI										**<0.001**
Low weight	434 (3.1)	297 (3.7)	68.4	20 (1.8)	4.6	104 (2.5)	24.0	13 (2.0)	3.0	
Normal weight	7,427 (53.1)	4,535 (56.5)	61.1	549 (50.0)	7.4	2043 (48.3)	27.5	300 (47.0)	4.0	
Overweight	5,285 (37.8)	2,806 (35.0)	53.1	453 (41.3)	8.6	1761 (41.6)	33.3	265 (41.5)	5.0	
Obesity	847 (6.1)	387 (4.8)	45.7	75 (6.8)	8.9	325 (7.7)	38.4	60 (9.4)	7.1	
Ethnicity										0.743
Han	13,988 (100.0)	8,021 (100.0)	57.3	1,097 (100.0)	7.8	4,232 (100.0)	30.3	638 (100.0)	4.6	
Others	5 (0.0)	4 (0.0)	80.0	0 (0.0)	0.0	1 (0.0)	20.0	0 (0.0)	0.0	
Education status										**0.017**
Illiteracy	1,645 (11.8)	958 (11.9)	58.2	104 (9.5)	6.3	517 (12.2)	31.4	66 (10.3)	4.0	
High school or below	11,886 (84.9)	6,781 (84.5)	57.1	950 (86.6)	8.0	3,601 (85.1)	30.3	554 (86.8)	4.7	
College or above	462 (3.3)	286 (3.6)	61.9	43 (3.9)	9.3	115 (2.7)	24.9	18 (2.8)	3.9	
Marital status										0.484
Unmarried	87 (0.6)	50 (0.6)	57.5	6 (0.5)	6.9	27 (0.6)	31.0	4 (0.6)	4.6	
Married	13,271 (94.8)	7,641 (95.2)	57.6	1,042 (95.0)	7.9	3,986 (94.2)	30.0	602 (94.4)	4.5	
Remarried	171 (1.2)	91 (1.1)	53.2	15 (1.4)	8.8	55 (1.3)	32.2	10 (1.6)	5.8	
Divorced or widowed	464 (3.3)	243 (3.0)	52.4	34 (3.1)	7.3	165 (3.9)	35.6	22 (3.4)	4.7	
Family history of CRC in first-degree relatives										**<0.001**
No	13,050 (93.3)	7,502 (93.5)	57.5	1,019 (92.9)	7.8	3,951 (93.3)	30.3	578 (90.6)	4.4	
Yes	412 (2.9)	188 (2.3)	45.6	27 (2.5)	6.6	164 (3.9)	39.8	33 (5.2)	8.0	
Unknown	531 (3.8)	335 (4.2)	63.1	51 (4.6)	9.6	118 (2.8)	22.2	27 (4.2)	5.1	
Family history of familial adenomatous polyposis in first-degree relatives										0.305
No	12,564 (89.8)	7,224 (90.0)	57.5	972 (88.6)	7.7	3,795 (89.7)	30.2	573 (89.8)	4.6	
Yes	369 (2.6)	217 (2.7)	58.8	37 (3.4)	10.0	97 (2.3)	26.3	18 (2.8)	4.9	
Unknown	1,060 (7.6)	584 (7.3)	55.1	88 (8.0)	8.3	341 (8.1)	32.2	47 (7.4)	4.4	
Cigarette smoking status										**<0.001**
Never	10,001 (71.5)	6,295 (78.4)	62.9	745 (67.9)	7.4	2,598 (61.4)	26.0	363 (56.9)	3.6	
Current	3,267 (23.3)	1,374 (17.1)	42.1	292 (26.6)	8.9	1,366 (32.3)	41.8	235 (36.8)	7.2	
Former	725 (5.2)	356 (4.4)	49.1	60 (5.5)	8.3	269 (6.4)	37.1	40 (6.3)	5.5	
Alcohol drinking status										**<0.001**
Never	10,103 (72.2)	6,302 (78.5)	62.4	780 (71.1)	7.7	2,635 (62.2)	26.1	386 (60.5)	3.8	
Current	3,670 (26.2)	1,614 (20.1)	44.0	303 (27.6)	8.3	1,511 (35.7)	41.2	242 (37.9)	6.6	
Former	220 (1.6)	109 (1.4)	49.5	14 (1.3)	6.4	87 (2.1)	39.5	10 (1.6)	4.5	
Frequency of physical activity										
Never	7,313 (52.3)	4,106 (51.2)	56.1	554 (50.5)	7.6	2,333 (55.1)	31.9	320 (50.2)	4.4	
1 ~ 3 times a month	1,237 (8.8)	794 (9.9)	64.2	107 (9.8)	8.6	295 (7.0)	23.8	41 (6.4)	3.3	
1 ~ 2 times a week	1894 (13.5)	1,209 (15.1)	63.8	139 (12.7)	7.3	462 (10.9)	24.4	84 (13.2)	4.4	
3 ~ 5 times a week	932 (6.7)	535 (6.7)	57.4	70 (6.4)	7.5	286 (6.8)	30.7	41 (6.4)	4.4	
Every day or almost every day	2,617 (18.7)	1,381 (17.2)	52.8	227 (20.7)	8.7	857 (20.2)	32.7	152 (23.8)	5.8	
Regular NSAID use										**<0.001**
No	13,418 (95.9)	7,750 (96.6)	57.8	1,055 (96.2)	7.9	4,010 (94.7)	29.9	603 (94.5)	4.5	
Yes	575 (4.1)	275 (3.4)	47.8	42 (3.8)	7.3	223 (5.3)	38.8	35 (5.5)	6.1	

Bold font indicates that the value is statistically significant after FDR correction (*p* < = 0.013).

### 3.1 Multivariable associations of lifestyle factors and dietary preference with SP and AD

As shown in Table 2, BMI was positively associated with the risk of both types of lesions. Compared to participants with normal weight, those with low weight had lower risks of only AD (OR = 0.78, 95%CI: 0.64–0.94), but those with overweight (OR = 1.22, 95%CI: 1.09–1.38 for only AD; OR = 1.18, 95%CI: 1.07–1.29 for only SP) or obesity (OR = 1.50, 95%CI: 1.23–1.84 for only SP, *p* for trend <0.001; OR = 1.53, 95%CI: 1.28–1.82 for only AD, *p* for trend <0.001; and OR = 1.97, 95%CI: 1.40–2.75 for synchronous SP and AD, *p* for trend =0.002) were more likely to develop AD and SP. Cigarette smoking also appeared to increase the risk of intestinal lesions. Compared to individuals who never smoked, current smokers had a 20–30% increased risk of only SP (OR = 1.29, 95%CI: 1.07–1.55) and only AD (OR = 1.24, 95%CI: 1.11–1.39), and an approximately 50% increased risk of synchronous SP and AD (OR = 1.53, 95%CI: 1.27–1.85). Further analysis found that compared to never smokers, current smokers who have been smoking for 31 to 40 years (OR = 1.45, 95%CI: 1.24–1.70 for only SP; OR = 1.31, 95%CI: 1.13–1.52 for only AD; OR = 1.44, 95%CI: 1.14–1.81 for synchronous SP and AD) or smoked more than 20 cigarettes per day (OR = 1.55, 95%CI: 1.17–2.06 for only SP; OR = 1.34, 95%CI: 1.15–1.57 for only AD; OR = 1.58, 95%CI: 1.19–2.09 for synchronous SP and AD) also significantly increased the risks of AD and SP. However, quitting smoking was shown to have a protective effect against polyps and adenomas. The OR of quitting smoking for ≤5 years was 0.53 for only SP (95%CI: 0.40–0.70), 0.58 for only AD (95%CI: 0.46–0.73) when compared to current smokers and the OR of quitting smoking for 6–12 years was 0.38 for synchronous SP and AD (95%CI: 0.24–0.61). As for alcohol drinking, current drinkers, drinking duration, and intensity were only observed to be positively associated with the risk of only AD (*p* for trend <0.001) but not only SP. In addition, no significant association between physical activity and regular aspirin use with SP and AD was observed. A negative association was observed between the frequency of whole-grain intake and the risk of only AD (e.g., OR = 0.85, 95%CI: 0.75–0.96 for eating whole grain 1–3 days per month vs. never eating whole grain), while the correlation of frequency of food intake and the two lesions was not observed in other categories.

**Table 2:** Association analysis of lifestyle factors and dietary preference with AD and SP.

Factors	No polyp control (*n* = 8,025)	Only serrated polyp (SP) (*n* = 1,097)			Only conventional adenoma (AD) (*n* = 4,233)			Synchronous SP and AD (*n* = 638)			*N* (%)	OR (95%CI)	*p* for trend	*N* (%)	OR (95%CI)	*p* for trend	*N* (%)	OR (95%CI)	*p* for trend
BMI ^a^										
Low weight	297 (3.7)	20 (1.8)	**0.60 (0.38–0.94)**	**<0.001**	104 (2.5)	**0.78 (0.64–0.94)**	**<0.001**	13 (2.0)	0.71 (0.39–1.29)	**0.002**
Normal weight	4,535 (56.5)	549 (50.0)	ref		2043 (48.3)	ref		300 (47.0)	ref	
Overweight	2,806 (35.0)	453 (41.3)	**1.22 (1.09–1.38)**		1761 (41.6)	**1.18 (1.07–1.29)**		265 (41.5)	1.20 (1.02–1.42)	
Obesity	387 (4.8)	75 (6.8)	**1.50 (1.23–1.84)**		325 (7.7)	**1.53 (1.28–1.82)**		60 (9.4)	**1.97 (1.40–2.75)**	
Cigarette smoking status ^b^										
Never	6,295 (78.4)	745 (67.9)	ref	-	2,598 (61.4)	ref	-	363 (56.9)	ref	-
Current	1,374 (17.1)	292 (26.6)	**1.29 (1.07–1.55)**		1,366 (32.3)	**1.24 (1.11–1.39)**		235 (36.8)	**1.53 (1.27–1.85)**	
Former	356 (4.4)	60 (5.5)	0.99 (0.75–1.31)		269 (6.4)	0.82 (0.67–1.01)		40 (6.3)	0.88 (0.68–1.14)	
Smoking duration, years ^b^										
Never	6,295 (78.4)	745 (67.9)	ref	0.034	2,598 (61.4)	ref	**<0.001**	363 (56.9)	ref	**<0.001**
≤30	716 (8.9)	134 (12.2)	1.14 (0.91–1.44)		631 (14.9)	1.17 (1.02–1.35)		97 (15.2)	1.29 (0.98–1.70)	
31–40	541 (6.7)	130 (11.9)	**1.45 (1.24–1.70)**		568 (13.4)	**1.31 (1.13–1.52)**		86 (13.5)	**1.44 (1.14–1.81)**	
>40	473 (5.9)	88 (8.0)	1.12 (0.77–1.64)		436 (10.3)	0.97 (0.83–1.13)		92 (14.4)	**1.50 (1.18–1.92)**	
Smoking intensity, pack/day ^b^										
Never	6,295 (78.4)	745 (67.9)	ref	**0.009**	2,598 (61.4)	ref	**<0.001**	363 (56.9)	ref	**<0.001**
≤15	651 (8.1)	124 (11.3)	1.13 (0.87–1.48)		616 (14.6)	1.12 (0.95–1.31)		97 (15.2)	1.25 (0.97–1.61)	
16–20	834 (10.4)	167 (15.2)	1.22 (0.99–1.52)		746 (17.6)	1.14 (1.02–1.27)		135 (21.2)	**1.47 (1.21–1.78)**	
>20	245 (3.1)	61 (5.6)	**1.55 (1.17–2.06)**		273 (6.4)	**1.34 (1.15–1.57)**		43 (6.7)	**1.58 (1.19–2.09)**	
Smoking cessation status, years ^b^										
Current	1,374 (79.4)	292 (83.0)	ref	0.463	1,366 (83.5)	ref	**<0.001**	235 (85.5)	ref	**<0.001**
Quit ≤5	133 (7.7)	15 (4.3)	**0.53 (0.40–0.70)**		86 (5.3)	**0.58 (0.46–0.73)**		18 (6.5)	0.71 (0.53–0.97)	
Quit 6–12	109 (6.3)	21 (6.0)	0.87 (0.58–1.31)		91 (5.6)	**0.75 (0.60–0.93)**		8 (2.9)	**0.38 (0.24–0.61)**	
Quit >12	114 (6.6)	24 (6.8)	0.94 (0.52–1.70)		92 (5.6)	**0.68 (0.52–0.87)**		14 (5.1)	0.60 (0.40–0.91)	
Alcohol drinking status ^c^										
Never	6,302 (78.5)	780 (71.1)	ref	-	2,635 (62.2)	ref	-	386 (60.5)	ref	-
Current	1,614 (20.1)	303 (27.6)	1.05 (0.81–1.35)		1,511 (35.7)	**1.28 (1.14–1.44)**		242 (37.9)	1.27 (0.96–1.69)	
Former	109 (1.4)	14 (1.3)	0.71 (0.36–1.40)		87 (2.1)	1.09 (0.85–1.41)		10 (1.6)	0.76 (0.48–1.19)	
Drinking duration, years ^c^										
Never	6,302 (78.5)	780 (71.1)	ref	0.603	2,635 (62.2)	ref	**<0.001**	386 (60.5)	ref	**0.001**
≤37	700 (8.7)	120 (10.9)	1.01 (0.70–1.45)		489 (11.6)	**1.21 (1.04–1.40)**		79 (12.4)	1.24 (0.80–1.92)	
38–45	540 (6.7)	106 (9.7)	1.05 (0.85–1.30)		566 (13.4)	**1.36 (1.12–1.66)**		82 (12.9)	1.17 (0.84–1.61)	
>45	483 (6.0)	91 (8.3)	1.03 (0.77–1.37)		543 (12.8)	**1.25 (1.12–1.40)**		91 (14.3)	1.34 (1.00–1.80)	
Alcohol intake, ml/week ^c^										
Never	6,302 (78.5)	780 (71.1)	ref	0.510	2,635 (62.2)	ref	**<0.001**	386 (60.5)	ref	0.021
≤25	651 (8.1)	109 (9.9)	0.99 (0.67–1.45)		494 (11.7)	1.19 (1.04–1.36)		80 (12.5)	1.18 (0.84–1.66)	
25.1–55.9	498 (6.2)	94 (8.6)	1.05 (0.75–1.46)		454 (10.7)	1.23 (1.02–1.49)		69 (10.8)	1.16 (0.88–1.54)	
>56	574 (7.2)	114 (10.4)	1.06 (0.85–1.33)		650 (15.4)	**1.41 (1.24–1.60)**		103 (16.1)	1.40 (1.04–1.87)	
Frequency of physical activity ^d^										
Never	4,106 (51.2)	554 (50.5)	ref	0.146	2,333 (55.1)	ref	0.239	320 (50.2)	ref	0.018
1 ~ 3 times a month	794 (9.9)	107 (9.8)	1.10 (0.86–1.39)		295 (7.0)	0.88 (0.71–1.10)		41 (6.4)	0.86 (0.60–1.23)	
1 ~ 2 times a week	1,209 (15.1)	139 (12.7)	0.95 (0.75–1.21)		462 (10.9)	0.90 (0.72–1.13)		84 (13.2)	1.22 (0.79–1.86)	
3 ~ 5 times a week	535 (6.7)	70 (6.4)	1.00 (0.78–1.30)		286 (6.8)	1.11 (0.93–1.33)		41 (6.4)	1.14 (0.81–1.58)	
Every day or almost every day	1,381 (17.2)	227 (20.7)	1.19 (0.99–1.42)		857 (20.2)	1.11 (0.93–1.32)		152 (23.8)	1.37 (1.03–1.81)	
Regular NSAID use ^e^										
No	7,750 (96.6)	1,055 (96.2)	ref	-	4,010 (94.7)	ref	-	603 (94.5)	ref	-
Yes	275 (3.4)	42 (3.8)	0.94 (0.74–1.19)		223 (5.3)	1.04 (0.89–1.22)		35 (5.5)	1.04 (0.78–1.38)	
Frequency of vegetable intake ^f^										
Never	51 (0.6)	4 (0.4)	0.50 (0.17–1.47)	0.566	28 (0.7)	1.52 (0.69–3.35)	0.080	2 (0.3)	0.43 (0.17–1.11)	0.448
1–3 days per month	112 (1.4)	16 (1.5)	ref		42 (1.0)	ref		10 (1.6)	ref	
1–3 days per week	542 (6.8)	61 (5.6)	0.68 (0.42–1.13)		222 (5.2)	1.05 (0.78–1.41)		32 (5.0)	0.57 (0.25–1.30)	
4–6 days per week	1,377 (17.2)	167 (15.2)	0.67 (0.40–1.13)		559 (13.2)	1.09 (0.77–1.54)		87 (13.6)	0.62 (0.34–1.12)	
Every day	5,943 (74.1)	849 (77.4)	0.73 (0.42–1.29)		3,382 (79.9)	1.25 (0.85–1.85)		507 (79.5)	0.69 (0.40–1.18)	
Frequency of fruit intake ^f^										
Never	267 (3.3)	41 (3.7)	ref	0.117	222 (5.2)	ref	0.490	35 (5.5)	ref	0.607
1–3 days per month	881 (11.0)	99 (9.0)	0.82 (0.60–1.12)		503 (11.9)	0.95 (0.72–1.24)		74 (11.6)	0.91 (0.62–1.35)	
1–3 days per week	2,739 (34.1)	359 (32.7)	0.97 (0.78–1.20)		1,452 (34.3)	0.95 (0.77–1.16)		201 (31.5)	0.79 (0.63–0.99)	
4–6 days per week	2,125 (26.5)	289 (26.3)	1.01 (0.77–1.31)		1,015 (24.0)	0.94 (0.78–1.14)		153 (24.0)	0.84 (0.62–1.12)	
Every day	2013 (25.1)	309 (28.2)	1.12 (0.83–1.51)		1,041 (24.6)	0.95 (0.74–1.22)		175 (27.4)	0.88 (0.66–1.17)	
Frequency of red meat intake ^f^										
Never	233 (2.9)	29 (2.6)	ref	0.198	123 (2.9)	ref	0.577	17 (2.7)	ref	0.033
1–3 days per month	1,266 (15.8)	131 (11.9)	0.76 (0.48–1.22)		568 (13.4)	1.04 (0.82–1.32)		59 (9.2)	0.67 (0.40–1.12)	
1–3 days per week	3,873 (48.3)	526 (47.9)	0.94 (0.60–1.47)		2084 (49.2)	1.17 (0.90–1.54)		312 (48.9)	1.08 (0.60–1.93)	
4–6 days per week	1803 (22.5)	291 (26.5)	1.14 (0.75–1.73)		981 (23.2)	1.20 (0.91–1.59)		161 (25.2)	1.12 (0.58–2.17)	
Every day	850 (10.6)	120 (10.9)	0.90 (0.55–1.47)		477 (11.3)	1.08 (0.74–1.57)		89 (13.9)	1.07 (0.59–1.92)	
Frequency of white meat intake ^f^										
Never	401 (5.0)	42 (3.8)	ref	0.812	254 (6.0)	ref	0.194	29 (4.5)	ref	0.105
1–3 days per month	1922 (24.0)	232 (21.1)	1.26 (0.85–1.87)		858 (20.3)	0.80 (0.63–1.01)		109 (17.1)	0.94 (0.49–1.80)	
1–3 days per week	4,043 (50.4)	599 (54.6)	1.39 (1.00–1.92)		2,204 (52.1)	0.91 (0.73–1.13)		341 (53.4)	1.17 (0.73–1.87)	
4–6 days per week	1,399 (17.4)	184 (16.8)	1.10 (0.76–1.61)		735 (17.4)	0.92 (0.79–1.07)		119 (18.7)	1.13 (0.74–1.71)	
Every day	260 (3.2)	40 (3.6)	1.21 (0.89–1.65)		182 (4.3)	1.10 (0.84–1.44)		40 (6.3)	1.56 (0.81–3.01)	
Frequency of bean and soy product intake ^f^										
Never	554 (6.9)	87 (7.9)	ref	0.657	382 (9.0)	ref	0.057	51 (8.0)	ref	0.887
1–3 days per month	2098 (26.1)	273 (24.9)	0.85 (0.62–1.14)		1,080 (25.5)	0.93 (0.81–1.06)		147 (23.0)	0.93 (0.66–1.29)	
1–3 days per week	3,973 (49.5)	542 (49.4)	0.84 (0.63–1.13)		2075 (49.0)	0.86 (0.75–0.98)		310 (48.6)	0.93 (0.67–1.30)	
4–6 days per week	1,160 (14.5)	153 (13.9)	0.84 (0.58–1.21)		556 (13.1)	0.80 (0.62–1.03)		98 (15.4)	1.00 (0.67–1.49)	
Every day	240 (3.0)	42 (3.8)	1.04 (0.70–1.55)		140 (3.3)	0.83 (0.60–1.15)		32 (5.0)	1.09 (0.46–2.56)	
Frequency of preserved vegetable intake ^f^										
Never	1,619 (20.2)	214 (19.5)	ref	0.406	726 (17.2)	ref	**0.006**	109 (17.1)	ref	0.497
1–3 days per month	3,280 (40.9)	478 (43.6)	1.13 (0.92–1.37)		1,637 (38.7)	1.13 (1.00–1.28)		262 (41.1)	1.15 (0.84–1.58)	
1–3 days per week	2,417 (30.1)	316 (28.8)	0.98 (0.81–1.18)		1,375 (32.5)	1.17 (0.99–1.37)		195 (30.6)	1.01 (0.79–1.30)	
4–6 days per week	514 (6.4)	65 (5.9)	0.92 (0.63–1.33)		352 (8.3)	1.32 (1.05–1.67)		42 (6.6)	0.92 (0.61–1.39)	
Every day	195 (2.4)	24 (2.2)	0.83 (0.48–1.43)		143 (3.4)	1.28 (1.02–1.60)		30 (4.7)	1.49 (0.92–2.40)	
Frequency of fried or grilled food intake ^f^										
Never	4,790 (59.7)	677 (61.7)	ref	0.182	2,705 (63.9)	ref	0.046	391 (61.3)	ref	0.362
1–3 days per month	2,316 (28.9)	318 (29.0)	1.00 (0.89–1.12)		1,146 (27.1)	1.01 (0.89–1.14)		180 (28.2)	1.05 (0.90–1.23)	
1–3 days per week	797 (9.9)	86 (7.8)	0.81 (0.61–1.09)		324 (7.7)	0.81 (0.66–1.00)		59 (9.2)	1.05 (0.77–1.43)	
4–6 days per week	99 (1.2)	13 (1.2)	1.08 (0.54–2.16)		49 (1.2)	1.05 (0.69–1.58)		6 (0.9)	0.84 (0.34–2.05)	
Every day	23 (0.3)	3 (0.3)	0.86 (0.27–2.74)		9 (0.2)	0.61 (0.31–1.21)		2 (0.3)	0.52 (0.15–1.86)	
Frequency of whole-grain intake ^f^										
Never	945 (11.8)	143 (13.0)	ref	0.407	624 (14.7)	ref	0.142	80 (12.5)	ref	0.885
1–3 days per month	2,299 (28.6)	321 (29.3)	0.96 (0.80–1.15)		1,149 (27.1)	**0.85 (0.75–0.96)**		198 (31.0)	1.15 (0.86–1.53)	
1–3 days per week	2,615 (32.6)	337 (30.7)	0.87 (0.74–1.03)		1,382 (32.6)	0.86 (0.72–1.03)		173 (27.1)	0.81 (0.59–1.11)	
4–6 days per week	1,107 (13.8)	147 (13.4)	0.89 (0.68–1.17)		570 (13.5)	0.86 (0.72–1.04)		91 (14.3)	1.00 (0.75–1.35)	
Every day	1,059 (13.2)	149 (13.6)	0.92 (0.76–1.12)		508 (12.0)	**0.78 (0.65–0.93)**		96 (15.0)	1.05 (0.68–1.62)	

^a^Adjusted for sex, age group, education status, family history of CRC or familial adenomatous polyposis in first-degree relatives, cigarette smoking status, alcohol drinking status, frequency of physical activity, regular NSAID use, and dietary preference. ^b^Adjusted for sex, age group, education status, family history of CRC or familial adenomatous polyposis in first-degree relatives, BMI, alcohol drinking status, frequency of physical activity, regular NSAID use, and dietary preference. ^c^Adjusted for sex, age group, education status, family history of CRC or familial adenomatous polyposis in first-degree relatives, BMI, cigarette smoking status, frequency of physical activity, regular NSAID use, and dietary preference. ^d^Adjusted for sex, age group, education status, family history of CRC or familial adenomatous polyposis in first-degree relatives, BMI, cigarette smoking status, alcohol drinking status, regular NSAID use, and dietary preference. ^e^Adjusted for sex, age group, education status, family history of CRC or familial adenomatous polyposis in first-degree relatives, BMI, cigarette smoking status, alcohol drinking status, frequency of physical activity, and dietary preference. ^f^Adjusted for sex, age group, education status, family history of CRC or familial adenomatous polyposis in first-degree relatives, BMI, cigarette smoking status, alcohol drinking status, frequency of physical activity, and regular NSAID use. Bold font indicates that the value is statistically significant after FDR correction (*p* < = 0.013).

To explore the influence of the number of these two lesions on the association between the factors mentioned above and the risk of only SP and only AD, we divided the SP and AD cases into one lesion and multiple lesion groups for analysis, and the results are shown in Table 3. The correlation between the most influence factors and the two lesions was consistent with the results in Table 2. Besides, regular NSAID use exhibited to reduce the risk of single SP (OR = 0.76, 95%CI: 0.63–0.92). In addition, a positive association was observed between the frequency of preserved vegetable intake and the risk of single SP (e.g., OR = 1.29, 95%CI: 1.07–1.56 for eating preserved vegetables 4–6 days per week vs. never eating preserved vegetables). However, there are some results that do not meet our expectations. Alcohol drinking for less than 37 years showed a protective effect on the risk of developing multiple SPs (OR = 0.51, 95%CI: 0.31–0.84), but in the current study, the sample size of this group was only 9 cases, so the results need to be verified by referring to literature with a larger sample size. Exercising more than 3 times a week (OR = 2.31, 95%CI: 1.39–3.83) and exercising every day (OR = 1.75, 95%CI: 1.29–2.38) increased the risk of developing multiple SPs.

**Table 3:** Association analysis of lifestyle factors and dietary preference with AD and SP according to the number of lesion.

Factors	No polyp Control	Serrated polyp (SP)							Conventional adenoma (AD)						
		The number of lesion site							The number of lesion site						
		1			>1			1			>1		
		*N* (%)	OR (95%CI)	*p* for trend	*N* (%)	OR (95%CI)	*p* for trend	*N* (%)	OR (95%CI)	*p* for trend	*N* (%)	OR (95%CI)	*p* for trend
BMI ^a^													
Low weight	297 (3.7)	19 (1.9)	0.63 (0.40–1.00)	**<0.001**	1 (0.8)	0.28 (0.04–2.10)	0.322	81 (2.7)	0.82 (0.69–0.99)	**<0.001**	23 (1.8)	0.64 (0.40–1.01)	**<0.001**
Normal weight	4,535 (56.5)	487 (49.8)	ref		62 (51.7)	ref		1,472 (49.4)	ref		571 (45.5)	ref	
Overweight	2,806 (35.0)	401 (41.0)	**1.24 (1.09–1.40)**		52 (43.3)	1.15 (0.86–1.53)		1,218 (40.9)	**1.16 (1.05–1.28)**		543 (43.3)	**1.22 (1.06–1.40)**	
Obesity	387 (4.8)	70 (7.2)	**1.61 (1.29–2.01)**		5 (4.2)	0.82 (0.36–1.90)		208 (7.0)	**1.42 (1.17–1.73)**		117 (9.3)	**1.83 (1.57–2.12)**	
Cigarette smoking status ^b^													
Never	6,295 (78.4)	679 (69.5)	ref	-	66 (55.0)	ref	-	1936 (65.0)	ref	-	662 (52.8)	ref	-
Current	1,374 (17.1)	245 (25.1)	1.20 (0.94–1.52)		47 (39.2)	**2.17 (1.38–3.42)**		865 (29.0)	**1.18 (1.06–1.31)**		501 (40.0)	1.36 (1.07–1.75)	
Former	356 (4.4)	53 (5.4)	0.98 (0.77–1.24)		7 (5.8)	1.14 (0.46–2.84)		178 (6.0)	0.82 (0.68–0.99)		91 (7.3)	0.82 (0.59–1.13)	
Smoking duration, years ^b^													
Never	6,295 (78.4)	679 (69.5)	ref	0.279	66 (55.0)	ref	**<0.001**	1936 (65.0)	ref	**<0.001**	662 (52.8)	ref	0.030
≤30	716 (8.9)	118 (12.1)	1.11 (0.88–1.40)		16 (13.3)	1.44 (0.87–2.38)		410 (13.8)	1.13 (1.01–1.27)		221 (17.6)	1.25 (0.95–1.66)	
31–40	541 (6.7)	103 (10.5)	1.27 (1.01–1.59)		27 (22.5)	**3.16 (1.56–6.43)**		356 (12.0)	**1.23 (1.08–1.40)**		212 (16.9)	1.48 (1.05–2.08)	
>40	473 (5.9)	77 (7.9)	1.10 (0.72–1.68)		11 (9.2)	1.39 (0.76–2.56)		277 (9.3)	0.94 (0.80–1.11)		159 (12.7)	1.01 (0.77–1.32)	
Smoking intensity, pack/day ^b^													
Never	6,295 (78.4)	679 (69.5)	ref	0.083	66 (55.0)	ref	**0.007**	1936 (65.0)	ref	**<0.001**	662 (52.8)	ref	**0.007**
≤15	651 (8.1)	104 (10.6)	1.06 (0.80–1.41)		20 (16.7)	**1.78 (1.13–2.80)**		377 (12.7)	1.04 (0.90–1.20)		239 (19.1)	1.28 (0.95–1.74)	
16–20	834 (10.4)	141 (14.4)	1.14 (0.87–1.49)		26 (21.7)	**2.02 (1.25–3.29)**		497 (16.7)	**1.13 (1.04–1.23)**		249 (19.9)	1.14 (0.89–1.46)	
>20	245 (3.1)	53 (5.4)	**1.46 (1.10–1.95)**		8 (6.7)	2.29 (0.93–5.68)		169 (5.7)	**1.24 (1.05–1.47)**		104 (8.3)	**1.52 (1.15–2.02)**	
Smoking cessation status, years ^b^													
Current	1,374 (79.4)	245 (82.2)	ref	0.644	47 (87.0)	ref	0.442	865 (82.9)	ref	0.040	501 (84.6)	ref	**0.001**
Quit ≤5	133 (7.7)	13 (4.4)	**0.55 (0.39–0.75)**		2 (3.7)	0.45 (0.14–1.44)		53 (5.1)	**0.56 (0.40–0.79)**		33 (5.6)	**0.60 (0.45–0.80)**	
Quit 6–12	109 (6.3)	20 (6.7)	1.00 (0.71–1.40)		1 (1.9)	0.25 (0.04–1.47)		62 (5.9)	0.81 (0.60–1.10)		29 (4.9)	0.65 (0.45–0.92)	
Quit >12	114 (6.6)	20 (6.7)	0.95 (0.54–1.69)		4 (7.4)	0.82 (0.19–3.59)		63 (6.0)	0.74 (0.57–0.95)		29 (4.9)	**0.56 (0.35–0.88)**	
Alcohol drinking status ^c^													
Never	6,302 (78.5)	696 (71.2)	ref	-	84 (70.0)	ref	-	1959 (65.8)	ref	-	676 (53.9)	ref	-
Current	1,614 (20.1)	267 (27.3)	1.09 (0.84–1.42)		36 (30.0)	0.77 (0.45–1.31)		959 (32.2)	**1.21 (1.09–1.35)**		552 (44.0)	**1.43 (1.21–1.68)**	
Former	109 (1.4)	14 (1.4)	0.84 (0.42–1.69)		0 (0.0)	-		61 (2.0)	1.15 (0.84–1.59)		26 (2.1)	0.97 (0.71–1.32)	
Drinking duration, years ^c^													
Never	6,302 (78.5)	696 (71.2)	ref	0.526	84 (70.0)	ref	0.920	1959 (65.8)	ref	**<0.001**	676 (53.9)	ref	**<0.001**
≤37	700 (8.7)	111 (11.4)	1.09 (0.75–1.59)		9 (7.5)	**0.51 (0.31–0.84)**		307 (10.3)	1.09 (0.93–1.29)		182 (14.5)	**1.50 (1.24–1.81)**	
38–45	540 (6.7)	92 (9.4)	1.08 (0.87–1.35)		14 (11.7)	0.80 (0.47–1.37)		374 (12.6)	**1.36 (1.12–1.65)**		192 (15.3)	**1.36 (1.07–1.71)**	
>45	483 (6.0)	78 (8.0)	1.05 (0.77–1.44)		13 (10.8)	0.89 (0.38–2.08)		339 (11.4)	**1.21 (1.07–1.36)**		204 (16.3)	**1.36 (1.19–1.56)**	
Alcohol intake, ml/week ^c^													
Never	6,302 (78.5)	696 (71.2)	ref	0.325	84 (70.0)	ref	0.203	1959 (65.8)	ref	**<0.001**	676 (53.9)	ref	**<0.001**
≤25	651 (8.1)	95 (9.7)	1.02 (0.70–1.47)		14 (11.7)	0.78 (0.39–1.55)		319 (10.7)	1.12 (0.99–1.26)		175 (14.0)	**1.35 (1.07–1.70)**	
25.1–55.9	498 (6.2)	83 (8.5)	1.10 (0.78–1.55)		11 (9.2)	0.74 (0.31–1.72)		294 (9.9)	1.20 (0.99–1.45)		160 (12.8)	1.32 (1.06–1.65)	
>56	574 (7.2)	103 (10.5)	1.14 (0.88–1.49)		11 (9.2)	0.63 (0.39–1.01)		407 (13.7)	**1.34 (1.17–1.53)**		243 (19.4)	**1.54 (1.31–1.81)**	
Frequency of physical activity ^d^													
Never	4,106 (51.2)	506 (51.8)	ref	0.447	48 (40.0)	ref	**0.001**	1,642 (55.1)	ref	0.724	691 (55.1)	ref	0.047
1 ~ 3 times a month	794 (9.9)	96 (9.8)	1.05 (0.81–1.37)		11 (9.2)	1.51 (0.95–2.42)		233 (7.8)	0.94 (0.74–1.20)		62 (4.9)	**0.69 (0.55–0.86)**	
1 ~ 2 times a week	1,209 (15.1)	128 (13.1)	0.95 (0.75–1.20)		11 (9.2)	0.94 (0.58–1.51)		351 (11.8)	0.93 (0.75–1.15)		111 (8.9)	0.81 (0.59–1.10)	
3 ~ 5 times a week	535 (6.7)	55 (5.6)	0.88 (0.70–1.11)		15 (12.5)	**2.31 (1.39–3.83)**		197 (6.6)	1.06 (0.86–1.32)		89 (7.1)	1.26 (0.96–1.65)	
Every day or almost every day	1,381 (17.2)	192 (19.7)	1.12 (0.93–1.36)		35 (29.2)	**1.75 (1.29–2.38)**		556 (18.7)	1.02 (0.87–1.20)		301 (24.0)	1.32 (1.00–1.74)	
Regular NSAID use ^e^													
No	7,750 (96.6)	947 (96.9)	ref	-	108 (90.0)	ref	-	2,830 (95.0)	ref	-	1,180 (94.1)	ref	-
Yes	275 (3.4)	30 (3.1)	**0.76 (0.63–0.92)**		12 (10.0)	2.14 (1.15–4.00)		149 (5.0)	1.05 (0.92–1.21)		74 (5.9)	1.02 (0.71–1.48)	
Frequency of vegetable intake ^f^													
Never	51 (0.6)	4 (0.4)	0.52 (0.17–1.59)	0.763	0 (0.0)	-	0.267	22 (0.7)	1.85 (0.80–4.30)	0.070	6 (0.5)	0.99 (0.35–2.81)	0.224
1–3 days per month	112 (1.4)	15 (1.5)	ref		1 (0.8)	ref		27 (0.9)	ref		15 (1.2)	ref	
1–3 days per week	542 (6.8)	55 (5.6)	0.65 (0.37–1.14)		6 (5.0)	1.01 (0.10–10.5)		158 (5.3)	1.13 (0.86–1.50)		64 (5.1)	0.89 (0.50–1.59)	
4–6 days per week	1,377 (17.2)	155 (15.9)	0.65 (0.37–1.16)		12 (10.0)	0.74 (0.08–6.95)		412 (13.8)	1.22 (0.91–1.62)		147 (11.7)	0.86 (0.46–1.61)	
Every day	5,943 (74.1)	748 (76.6)	0.70 (0.38–1.27)		101 (84.2)	1.06 (0.13–8.29)		2,360 (79.2)	1.40 (1.03–1.91)		1,022 (81.5)	1.00 (0.50–2.00)	
Frequency of fruit intake ^f^													
Never	267 (3.3)	37 (3.8)	ref	0.185	4 (3.3)	ref	0.368	137 (4.6)	ref	0.914	85 (6.8)	ref	0.160
1–3 days per month	881 (11.0)	88 (9.0)	0.80 (0.58–1.10)		11 (9.2)	1.03 (0.40–2.64)		345 (11.6)	1.00 (0.77–1.31)		158 (12.6)	0.87 (0.61–1.24)	
1–3 days per week	2,739 (34.1)	327 (33.5)	0.98 (0.76–1.27)		32 (26.7)	0.85 (0.46–1.56)		1,041 (34.9)	1.05 (0.84–1.31)		411 (32.8)	0.78 (0.61–1.00)	
4–6 days per week	2,125 (26.5)	262 (26.8)	1.02 (0.73–1.42)		27 (22.5)	0.95 (0.51–1.76)		737 (24.7)	1.04 (0.85–1.29)		278 (22.2)	0.78 (0.62–0.97)	
Every day	2013 (25.1)	263 (26.9)	1.08 (0.77–1.52)		46 (38.3)	1.54 (0.64–3.71)		719 (24.1)	1.03 (0.80–1.32)		322 (25.7)	0.84 (0.61–1.15)	
Frequency of red meat intake ^f^													
Never	233 (2.9)	27 (2.8)	ref	0.255	2 (1.7)	ref	0.191	92 (3.1)	ref	0.977	31 (2.5)	ref	0.269
1–3 days per month	1,266 (15.8)	124 (12.7)	0.76 (0.46–1.27)		7 (5.8)	0.63 (0.12–3.36)		417 (14.0)	0.97 (0.80–1.16)		151 (12.0)	1.22 (0.79–1.88)	
1–3 days per week	3,873 (48.3)	467 (47.8)	0.89 (0.58–1.35)		59 (49.2)	1.57 (0.28–8.71)		1,496 (50.2)	1.09 (0.89–1.34)		588 (46.9)	1.36 (0.88–2.12)	
4–6 days per week	1803 (22.5)	250 (25.6)	1.05 (0.69–1.60)		41 (34.2)	2.33 (0.47–11.67)		654 (22.0)	1.05 (0.85–1.31)		327 (26.1)	1.62 (1.04–2.52)	
Every day	850 (10.6)	109 (11.2)	0.89 (0.56–1.42)		11 (9.2)	1.01 (0.21–4.97)		320 (10.7)	0.99 (0.74–1.33)		157 (12.5)	1.29 (0.73–2.28)	
Frequency of white meat intake ^f^													
Never	401 (5.0)	38 (3.9)	ref	0.733	4 (3.3)	ref	0.768	177 (5.9)	ref	0.772	77 (6.1)	ref	0.029
1–3 days per month	1922 (24.0)	212 (21.7)	1.27 (0.86–1.88)		20 (16.7)	1.27 (0.29–5.60)		648 (21.8)	0.85 (0.64–1.12)		210 (16.7)	0.69 (0.48–0.99)	
1–3 days per week	4,043 (50.4)	532 (54.5)	1.40 (1.00–1.96)		67 (55.8)	1.29 (0.36–4.58)		1,550 (52.0)	0.91 (0.72–1.15)		654 (52.2)	0.91 (0.67–1.23)	
4–6 days per week	1,399 (17.4)	159 (16.3)	1.11 (0.76–1.62)		25 (20.8)	1.06 (0.34–3.31)		493 (16.5)	0.89 (0.77–1.05)		242 (19.3)	0.98 (0.75–1.28)	
Every day	260 (3.2)	36 (3.7)	1.27 (0.95–1.70)		4 (3.3)	0.82 (0.20–3.30)		111 (3.7)	1.00 (0.71–1.43)		71 (5.7)	1.29 (0.87–1.91)	
Frequency of bean and soy product intake ^f^													
Never	554 (6.9)	80 (8.2)	ref	0.372	7 (5.8)	ref	0.221	260 (8.7)	ref	0.058	122 (9.7)	ref	0.078
1–3 days per month	2098 (26.1)	249 (25.5)	0.82 (0.57–1.18)		24 (20.0)	1.09 (0.55–2.14)		788 (26.5)	0.94 (0.79–1.12)		292 (23.3)	0.88 (0.69–1.11)	
1–3 days per week	3,973 (49.5)	480 (49.1)	0.81 (0.57–1.16)		62 (51.7)	1.18 (0.55–2.51)		1,449 (48.6)	0.85 (0.73–0.99)		626 (49.9)	0.85 (0.74–0.98)	
4–6 days per week	1,160 (14.5)	134 (13.7)	0.81 (0.53–1.23)		19 (15.8)	1.21 (0.56–2.65)		392 (13.2)	0.82 (0.64–1.05)		164 (13.1)	0.74 (0.56–1.00)	
Every day	240 (3.0)	34 (3.5)	0.92 (0.62–1.36)		8 (6.7)	2.31 (0.82–6.47)		90 (3.0)	0.83 (0.57–1.21)		50 (4.0)	0.84 (0.59–1.19)	
Frequency of preserved vegetable intake ^f^													
Never	1,619 (20.2)	191 (19.5)	ref	0.462	23 (19.2)	ref	0.393	526 (17.7)	ref	0.017	200 (15.9)	ref	**0.003**
1–3 days per month	3,280 (40.9)	430 (44.0)	1.13 (0.93–1.38)		48 (40.0)	1.09 (0.71–1.67)		1,180 (39.6)	1.13 (1.01–1.26)		457 (36.4)	1.15 (0.94–1.42)	
1–3 days per week	2,417 (30.1)	275 (28.1)	0.97 (0.79–1.19)		41 (34.2)	1.09 (0.67–1.78)		946 (31.8)	1.15 (0.98–1.35)		429 (34.2)	1.22 (0.98–1.53)	
4–6 days per week	514 (6.4)	59 (6.0)	0.95 (0.66–1.37)		6 (5.0)	0.70 (0.29–1.73)		236 (7.9)	**1.29 (1.07–1.56)**		116 (9.3)	1.39 (0.96–2.02)	
Every day	195 (2.4)	22 (2.3)	0.88 (0.54–1.42)		2 (1.7)	0.55 (0.1–3.07)		91 (3.1)	1.24 (0.98–1.57)		52 (4.1)	1.37 (1.01–1.86)	
Frequency of fried or grilled food intake ^f^													
Never	4,790 (59.7)	602 (61.6)	ref	0.242	75 (62.5)	ref	0.890	1891 (63.5)	ref	0.038	814 (64.9)	ref	0.118
1–3 days per month	2,316 (28.9)	288 (29.5)	1.00 (0.89–1.12)		30 (25.0)	0.96 (0.61–1.51)		828 (27.8)	1.01 (0.91–1.14)		318 (25.4)	1.00 (0.80–1.24)	
1–3 days per week	797 (9.9)	72 (7.4)	0.76 (0.51–1.11)		14 (11.7)	1.33 (0.69–2.56)		225 (7.6)	0.80 (0.63–1.01)		99 (7.9)	0.88 (0.64–1.20)	
4–6 days per week	99 (1.2)	12 (1.2)	1.09 (0.53–2.27)		1 (0.8)	0.87 (0.08–9.81)		31 (1.0)	0.94 (0.61–1.45)		18 (1.4)	1.35 (0.86–2.13)	
Every day	23 (0.3)	3 (0.3)	1.00 (0.33–3.03)		0 (0.0)	-		4 (0.1)	0.41 (0.14–1.21)		5 (0.4)	0.96 (0.54–1.72)	
Frequency of whole-grain intake ^f^													
Never	945 (11.8)	126 (12.9)	ref	0.269	17 (14.2)	ref	0.629	430 (14.4)	ref	0.347	194 (15.5)	ref	0.104
1–3 days per month	2,299 (28.6)	294 (30.1)	0.99 (0.81–1.22)		27 (22.5)	0.70 (0.38–1.30)		808 (27.1)	0.84 (0.72–0.99)		341 (27.2)	0.88 (0.71–1.10)	
1–3 days per week	2,615 (32.6)	299 (30.6)	0.90 (0.74–1.09)		38 (31.7)	0.74 (0.50–1.11)		982 (33.0)	0.89 (0.71–1.11)		400 (31.9)	**0.83 (0.74–0.93)**	
4–6 days per week	1,107 (13.8)	133 (13.6)	0.93 (0.73–1.20)		14 (11.7)	0.61 (0.27–1.35)		403 (13.5)	0.89 (0.69–1.15)		167 (13.3)	0.82 (0.63–1.08)	
Every day	1,059 (13.2)	125 (12.8)	0.90 (0.74–1.09)		24 (20.0)	1.02 (0.69–1.52)		356 (12.0)	0.80 (0.65–0.99)		152 (12.1)	0.74 (0.58–0.94)	

^a^Adjusted for sex, age group, education status, family history of CRC or familial adenomatous polyposis in first-degree relatives, cigarette smoking status, alcohol drinking status, frequency of physical activity, regular NSAID use, and dietary preference. ^b^Adjusted for sex, age group, education status, family history of CRC or familial adenomatous polyposis in first-degree relatives, BMI, alcohol drinking status, frequency of physical activity, regular NSAID use, and dietary preference. ^c^Adjusted for sex, age group, education status, family history of CRC or familial adenomatous polyposis in first-degree relatives, BMI, cigarette smoking status, frequency of physical activity, regular NSAID use, and dietary preference. ^d^Adjusted for sex, age group, education status, family history of CRC or familial adenomatous polyposis in first-degree relatives, BMI, cigarette smoking status, alcohol drinking status, regular NSAID use, and dietary preference. ^e^Adjusted for sex, age group, education status, family history of CRC or familial adenomatous polyposis in first-degree relatives, BMI, cigarette smoking status, alcohol drinking status, frequency of physical activity, and dietary preference. ^f^Adjusted for sex, age group, education status, family history of CRC or familial adenomatous polyposis in first-degree relatives, BMI, cigarette smoking status, alcohol drinking status, frequency of physical activity, and regular NSAID use. Bold font indicates that the value is statistically significant after FDR correction (*p* < = 0.013).

### 3.2 Multivariable associations of lifestyle factors and dietary preference with SP and AD in case-group comparison

As shown in Table 4, with only AD cases as reference, the smoking duration was associated with an increased risk of synchronous SP and AD (OR = 1.52, 95%CI: 1.20–1.93 for smoking more than 40 years vs. never smoking), and quitting smoking between 6 and 12 years reduced the risk of synchronous SP and AD (OR = 0.51, 95%CI: 0.32–0.81).

**Table 4:** Association analysis of lifestyle factors and dietary preference with AD and SP in case-group comparisons.

Factors	Only conventional adenoma (AD) control (*n* = 4,233)	Only serrated polyp (SP) (*n* = 1,097)			Synchronous SP and AD (*n* = 638)			*N* (%)	OR (95%CI)	*p* for trend	*N* (%)	OR (95%CI)	*p* for trend
BMI ^a^							
Low weight	104 (2.5)	20 (1.8)	0.78 (0.51–1.18)	0.480	13 (2.0)	0.95 (0.54–1.66)	0.330
Normal weight	2043 (48.3)	549 (50.0)	ref		300 (47.0)	ref	
Overweight	1761 (41.6)	453 (41.3)	1.03 (0.93–1.15)		265 (41.5)	1.02 (0.90–1.17)	
Obesity	325 (7.7)	75 (6.8)	0.97 (0.81–1.16)		60 (9.4)	1.30 (0.93–1.80)	
Cigarette smoking status ^b^							
Never	2,598 (61.4)	745 (67.9)	ref	-	363 (56.9)	ref	-
Current	1,366 (32.3)	292 (26.6)	1.02 (0.82–1.26)		235 (36.8)	1.21 (1.02–1.44)	
Former	269 (6.4)	60 (5.5)	1.19 (0.88–1.61)		40 (6.3)	1.05 (0.91–1.22)	
Smoking duration, years ^b^							
Never	2,598 (61.4)	745 (67.9)	ref	0.958	363 (56.9)	ref	**0.002**
≤30	631 (14.9)	134 (12.2)	0.95 (0.79–1.15)		97 (15.2)	1.09 (0.84–1.41)	
31–40	568 (13.4)	130 (11.9)	1.09 (0.88–1.35)		86 (13.5)	1.08 (0.85–1.35)	
>40	436 (10.3)	88 (8.0)	1.16 (0.76–1.76)		92 (14.4)	**1.52 (1.20–1.93)**	
Smoking intensity, pack/day ^b^							
Never	2,598 (61.4)	745 (67.9)	ref	0.770	363 (56.9)	ref	0.062
≤15	616 (14.6)	124 (11.3)	1.00 (0.74–1.35)		97 (15.2)	1.11 (0.91–1.35)	
16–20	746 (17.6)	167 (15.2)	1.05 (0.82–1.35)		135 (21.2)	1.26 (1.04–1.53)	
>20	273 (6.4)	61 (5.6)	1.13 (0.83–1.54)		43 (6.7)	1.16 (0.87–1.54)	
Smoking cessation status, years ^b^							
Current	1,366 (83.5)	292 (83.0)	ref	0.452	235 (85.5)	ref	**0.012**
Quit ≤5	86 (5.3)	15 (4.3)	0.93 (0.67–1.29)		18 (6.5)	1.20 (0.99–1.46)	
Quit 6–12	91 (5.6)	21 (6.0)	1.16 (0.67–1.99)		8 (2.9)	**0.51 (0.32–0.81)**	
Quit >12	92 (5.6)	24 (6.8)	1.40 (0.87–2.25)		14 (5.1)	0.90 (0.72–1.12)	
Alcohol drinking status ^c^							
Never	2,635 (62.2)	780 (71.1)	ref	-	386 (60.5)	ref	-
Current	1,511 (35.7)	303 (27.6)	0.83 (0.66–1.06)		242 (37.9)	1.00 (0.76–1.31)	
Former	87 (2.1)	14 (1.3)	0.65 (0.38–1.13)		10 (1.6)	0.71 (0.45–1.11)	
Drinking duration, years ^c^							
Never	2,635 (62.2)	780 (71.1)	ref	0.032	386 (60.5)	ref	0.975
≤37	489 (11.6)	120 (10.9)	0.84 (0.61–1.17)		79 (12.4)	1.02 (0.70–1.49)	
38–45	566 (13.4)	106 (9.7)	0.79 (0.61–1.03)		82 (12.9)	0.85 (0.65–1.11)	
>45	543 (12.8)	91 (8.3)	0.84 (0.63–1.10)		91 (14.3)	1.09 (0.77–1.53)	
Alcohol intake, ml/week ^c^							
Never	2,635 (62.2)	780 (71.1)	ref	0.026	386 (60.5)	ref	0.826
≤25	494 (11.7)	109 (9.9)	0.85 (0.60–1.19)		80 (12.5)	1.00 (0.75–1.35)	
25.1–55.9	454 (10.7)	94 (8.6)	0.86 (0.61–1.23)		69 (10.8)	0.94 (0.73–1.22)	
>56	650 (15.4)	114 (10.4)	0.77 (0.61–0.97)		103 (16.1)	0.99 (0.71–1.37)	
Frequency of physical activity ^d^							
Never	2,333 (55.1)	554 (50.5)	ref	0.694	320 (50.2)	ref	0.024
1 ~ 3 times a month	295 (7.0)	107 (9.8)	1.24 (0.87–1.78)		41 (6.4)	0.94 (0.65–1.36)	
1 ~ 2 times a week	462 (10.9)	139 (12.7)	1.06 (0.85–1.33)		84 (13.2)	1.32 (0.94–1.84)	
3 ~ 5 times a week	286 (6.8)	70 (6.4)	0.92 (0.71–1.20)		41 (6.4)	1.01 (0.69–1.49)	
Every day or almost every day	857 (20.2)	227 (20.7)	1.08 (0.90–1.30)		152 (23.8)	1.24 (0.98–1.56)	
Regular NSAID use ^e^							
No	4,010 (94.7)	1,055 (96.2)	ref	-	603 (94.5)	ref	-
Yes	223 (5.3)	42 (3.8)	0.91 (0.72–1.14)		35 (5.5)	1.01 (0.73–1.41)	
Frequency of vegetable intake ^f^							
Never	28 (0.7)	4 (0.4)	**0.33 (0.15–0.75)**	0.279	2 (0.3)	**0.32 (0.14–0.71)**	0.645
1–3 days per month	42 (1.0)	16 (1.5)	ref		10 (1.6)	ref	
1–3 days per week	222 (5.2)	61 (5.6)	0.63 (0.36–1.10)		32 (5.0)	0.61 (0.25–1.52)	
4–6 days per week	559 (13.2)	167 (15.2)	0.61 (0.33–1.16)		87 (13.6)	0.63 (0.29–1.38)	
Every day	3,382 (79.9)	849 (77.4)	0.59 (0.31–1.10)		507 (79.5)	0.61 (0.29–1.25)	
Frequency of fruit intake ^f^							
Never	222 (5.2)	41 (3.7)	ref	**0.011**	35 (5.5)	ref	0.852
1–3 days per month	503 (11.9)	99 (9.0)	0.87 (0.57–1.31)		74 (11.6)	0.98 (0.68–1.40)	
1–3 days per week	1,452 (34.3)	359 (32.7)	1.03 (0.72–1.45)		201 (31.5)	0.85 (0.67–1.08)	
4–6 days per week	1,015 (24.0)	289 (26.3)	1.07 (0.73–1.57)		153 (24.0)	0.92 (0.68–1.25)	
Every day	1,041 (24.6)	309 (28.2)	1.21 (0.85–1.72)		175 (27.4)	0.95 (0.69–1.31)	
Frequency of red meat intake ^f^							
Never	123 (2.9)	29 (2.6)	ref	0.351	17 (2.7)	ref	0.067
1–3 days per month	568 (13.4)	131 (11.9)	0.73 (0.45–1.19)		59 (9.2)	0.62 (0.33–1.16)	
1–3 days per week	2084 (49.2)	526 (47.9)	0.77 (0.53–1.12)		312 (48.9)	0.87 (0.47–1.61)	
4–6 days per week	981 (23.2)	291 (26.5)	0.92 (0.66–1.28)		161 (25.2)	0.88 (0.40–1.92)	
Every day	477 (11.3)	120 (10.9)	0.80 (0.58–1.10)		89 (13.9)	0.94 (0.52–1.72)	
Frequency of white meat intake ^f^							
Never	254 (6.0)	42 (3.8)	ref	0.403	29 (4.5)	ref	0.229
1–3 days per month	858 (20.3)	232 (21.1)	**1.60 (1.15–2.23)**		109 (17.1)	1.16 (0.63–2.14)	
1–3 days per week	2,204 (52.1)	599 (54.6)	**1.56 (1.18–2.06)**		341 (53.4)	1.30 (0.85–1.99)	
4–6 days per week	735 (17.4)	184 (16.8)	1.21 (0.82–1.78)		119 (18.7)	1.24 (0.83–1.85)	
Every day	182 (4.3)	40 (3.6)	1.10 (0.80–1.51)		40 (6.3)	1.45 (0.73–2.87)	
Frequency of bean and soy product intake ^f^							
Never	382 (9.0)	87 (7.9)	ref	0.445	51 (8.0)	ref	0.402
1–3 days per month	1,080 (25.5)	273 (24.9)	0.92 (0.68–1.24)		147 (23.0)	1.02 (0.74–1.41)	
1–3 days per week	2075 (49.0)	542 (49.4)	0.98 (0.71–1.34)		310 (48.6)	1.09 (0.74–1.62)	
4–6 days per week	556 (13.1)	153 (13.9)	1.05 (0.63–1.74)		98 (15.4)	1.26 (0.73–2.17)	
Every day	140 (3.3)	42 (3.8)	1.21 (0.80–1.83)		32 (5.0)	1.34 (0.64–2.78)	
Frequency of preserved vegetable intake ^f^							
Never	726 (17.2)	214 (19.5)	ref	0.059	109 (17.1)	ref	0.323
1–3 days per month	1,637 (38.7)	478 (43.6)	0.99 (0.82–1.19)		262 (41.1)	1.01 (0.75–1.36)	
1–3 days per week	1,375 (32.5)	316 (28.8)	0.84 (0.67–1.06)		195 (30.6)	0.87 (0.64–1.19)	
4–6 days per week	352 (8.3)	65 (5.9)	0.68 (0.39–1.18)		42 (6.6)	0.69 (0.46–1.03)	
Every day	143 (3.4)	24 (2.2)	0.67 (0.37–1.23)		30 (4.7)	1.15 (0.72–1.85)	
Frequency of fried or grilled food intake ^f^							
Never	2,705 (63.9)	677 (61.7)	ref	0.385	391 (61.3)	ref	0.352
1–3 days per month	1,146 (27.1)	318 (29.0)	0.99 (0.85–1.14)		180 (28.2)	1.05 (0.92–1.19)	
1–3 days per week	324 (7.7)	86 (7.8)	1.03 (0.81–1.31)		59 (9.2)	1.29 (0.97–1.73)	
4–6 days per week	49 (1.2)	13 (1.2)	1.04 (0.49–2.23)		6 (0.9)	0.82 (0.30–2.26)	
Every day	9 (0.2)	3 (0.3)	1.51 (0.38–5.95)		2 (0.3)	0.92 (0.31–2.73)	
Frequency of whole-grain intake ^f^							
Never	624 (14.7)	143 (13.0)	ref	0.546	80 (12.5)	ref	0.563
1–3 days per month	1,149 (27.1)	321 (29.3)	1.13 (0.92–1.39)		198 (31.0)	1.38 (0.99–1.92)	
1–3 days per week	1,382 (32.6)	337 (30.7)	1.02 (0.81–1.30)		173 (27.1)	0.96 (0.66–1.38)	
4–6 days per week	570 (13.5)	147 (13.4)	1.05 (0.81–1.35)		91 (14.3)	1.18 (0.79–1.77)	
Every day	508 (12.0)	149 (13.6)	1.21 (0.99–1.46)		96 (15.0)	1.36 (0.92–2.00)	

^a^Adjusted for sex, age group, education status, family history of CRC or familial adenomatous polyposis in first-degree relatives, cigarette smoking status, alcohol drinking status, frequency of physical activity, regular NSAID use, and dietary preference. ^b^Adjusted for sex, age group, education status, family history of CRC or familial adenomatous polyposis in first-degree relatives, BMI, alcohol drinking status, frequency of physical activity, regular NSAID use, and dietary preference. ^c^Adjusted for sex, age group, education status, family history of CRC or familial adenomatous polyposis in first-degree relatives, BMI, cigarette smoking status, frequency of physical activity, regular NSAID use, and dietary preference. ^d^Adjusted for sex, age group, education status, family history of CRC or familial adenomatous polyposis in first-degree relatives, BMI, cigarette smoking status, alcohol drinking status, regular NSAID use, and dietary preference. ^e^Adjusted for sex, age group, education status, family history of CRC or familial adenomatous polyposis in first-degree relatives, BMI, cigarette smoking status, alcohol drinking status, frequency of physical activity, and dietary preference. ^f^Adjusted for sex, age group, education status, family history of CRC or familial adenomatous polyposis in first-degree relatives, BMI, cigarette smoking status, alcohol drinking status, frequency of physical activity, and regular NSAID use.

As for dietary preference, although statistically significant results were observed between the only SP/synchronous AD and SP risk and vegetable intake, the sample size of the group who never ate vegetables was too small to be credible. The white meat intake was more likely to develop serrated polyp than conventional adenoma (e.g., OR = 1.60, 95%CI: 1.15–2.23 for eating 1–3 days per month vs. never eating). No association was found between other factors and SP or synchronous SP and AD when compared with only AD cases.

## 4 Discussion

This study was intended to assess the relationship between modifiable lifestyle factors and serrated polyp and conventional adenoma. We found that BMI and cigarette smoking were significantly associated with the increased risk of only SP, only AD, and synchronous SP and AD, whereas alcohol drinking showed a positive correlation with the risk of only AD. The case-group comparison indicated that cigarette smoking was more strongly associated with synchronous SP and AD than only AD. Besides, in the analysis of influencing factors related to dietary preference, the consumption of whole grains was inversely associated with the risk of only AD. In case-group comparison, the consumption of white meat was found to be positively associated with only SP than only AD.

Previous studies have demonstrated that obesity is an established risk factor for colorectal cancer (12). Similarly, positive associations between BMI and SP/AD have been reported in some literature (20, 21). Our study also supported this correlation. The inflammatory state of adipose tissue in the condition of obesity creates a favorable environment for tumor development (22). For example, the excess cytokines and adipokines produced by the increased adipose tissue will activate a series of signaling pathways, including phosphoinositide kinase-3 (PI3K)/serine–threonine protein kinase (AKT), leading to hyperplasia, proliferation, and carcinogenesis in colon cells (23). Furthermore, the distinguishing traits of the serrated-neoplasia pathway included the mutation of the BRAF gene, CpG island methylation phenotype (CIMP), and DNA microsatellite instability (MSI) (5, 8, 24). A chronic inflammatory environment can also induce microsatellite instability (MSI) by downregulating DNA repair pathways, resulting in the development of lesions in the serrated pathway (25). However, unlike previous research reports (15, 25), no stronger association between BMI and serrated polyps than traditional adenomas was observed in this study. Besides, physical activity, which was important in obesity-related cancers, was found to decrease colorectal cancer risk in previous studies, especially high levels of physical activity (26). However, in this analysis, people who exercised almost every day had a higher risk of multiple SPs and ADs. We speculate that this is due to the higher proportion of older people in the screening cohort. When we stratified the population by age, in a multivariate analysis, we found that participants aged ≥ 60 years who exercised daily or almost daily had a higher risk of developing SP (OR = 1.30, 95%CI: 1.13–1.49) or synchronous SP and AD (OR = 1.44, 95%CI: 1.11–1.87) than those who never exercised, but no such association was found in people aged <60 years (*p* > 0.05). Therefore, the reason for the higher risk among residents who exercised almost every day in our analysis could be due to confounding bias brought on by age. We suspect that people over the age of 60 may increase their exercise frequency because they already felt in poor physical condition themselves, but the short-term increase in exercise frequency did not reduce their risk of disease.

While only AD was taken as the control group, this study found that smoking appeared to be more strongly associated with the risk of synchronous SP and AD, which was consistent with the previous findings. Smoking has been found to have differential effects on different molecular pathways in CRC (27, 28). Current smoking showed higher ORs for MSI-high, CIMP-high, and BRAF-mutated subtype CRC, suggesting that smoking was more strongly associated with serrated pathway (27, 29). The analysis of the association between CRC subtype risk and other smoking-related variables including density and total duration reported the same results (29). Therefore, epigenetic alterations may contribute to smoking-induced colorectal neoplasms (12). Interestingly, Sonja et al. (30) reported an observation that there were widespread changes in DNA methylation patterns in smokers compared with never smokers. In former smokers, methylation levels were found to be similar to those in never smokers, suggesting that quitting smoking could restore aberrant methylation to normal levels (30). The protective effect of quitting smoking found in this study could confirm the study mentioned above. In addition, as the positive correlation between alcohol consumption and CRC was reported in many previous studies (31–33), alcohol drinking was a well-established risk factor for cancer. However, the pathways of alcohol to cancer development were not fully understood (34). According to related studies (13, 27, 35), there were no major differences were observed in the relationship between alcohol and CRC subtypes, but some studies (36) have indicated that alcohol intake was associated with an increased risk of traditional adenoma–carcinoma pathway but not serrated pathway. The results of our analysis support this finding.

Dietary fiber was generally thought to be beneficial for colorectal cancer. However, due to the fact that dietary fiber actually contains a wide range of materials, the population research results were not consistent (14, 37–40). He et al. (40) indicated that higher whole-grain intake was associated with lower CRC risk in men but not in women, and no heterogeneity was detected by subtypes. In this study, we found that whole grains were negatively associated with the traditional adenomas, but not serrated polyps. Although this may have been a chance finding, some studies (40) have also shown that fiber intake was not associated with mutation states such as BRAF, CIMP, and MSI that are highly associated with SP. However, other studies have reported different results. For example, Martha et al. (41) reported that higher dietary fiber was associated with a reduced risk of having a CIMP-mutated or a BRAF-mutated tumor. Similarly, white meat has also been inconsistently associated with CRC risk. A previous study conducted in Lanxi, China, reported that both poultry and seafood consumption were negatively associated with colorectal polyps (42). Another systematic review found a mild inverse association between fish and CRC risk, but less evidence for white meat (43). Therefore, future studies on white meat may need to classify it as either seafood sources or poultry sources to better explore its impact on CRC risk.

This is a large-scale population-based case–control study with an advantage in sample size. The colonoscopy examination of the subjects was conducted in designated hospitals, so a more consistent examination procedure and evaluation criteria were used to eliminate some investigation bias. In addition, the questionnaire information including socio-demographics, disease history, lifestyle, and so on was collected by trained staff through face-to-face questioning, thus having better credibility. There are also several weaknesses within this study. First, participants were asked to recall lifestyle information from the past year and report their anthropometric information, disease history, and so on; in addition to recall bias, the data, which was entirely self-reported by participants, can also be a source of bias. Meanwhile, compared with the course of CRC, the exposure assessed in this article was basically at the same time point as the outcome, so there may be limitations in inferring the long-term effects of factors such as physical activity and dietary preferences. Besides, the included population in the study were those who chose to undergo diagnostic screening who were at high CRC risk, while those who did not attend colonoscopy were not in the analysis. People who volunteer to undergo screening may place more emphasis on managing their own health; thus, it may result in an impact on the evaluation results. However, in all the people who underwent colonoscopy and had definite diagnosis, this study excluded a number of patients with non-adenomatous polyps, accounting for approximately 2% of the total number. The absence of data on this segment of the population may also have affected our results.

In summary, our assessment found common risk factors for SP and AD, such as high body mass index and a history of smoking, while alcohol consumption was more strongly associated with the risk of AD, suggesting that lifestyle changes should be made to reduce the risk of colorectal cancer. In addition, in terms of dietary factors, most of the influences on the risk of AD and SP have not been clarified. In future, more in-depth analysis can be conducted based on the detailed classification of different dietary sources or the comprehensive consideration of various diets.

## Data availability statement

The original contributions presented in the study are included in the article/Supplementary material, further inquiries can be directed to the corresponding author.

## Ethics statement

The studies involving humans were approved by Ethics review committee of Hangzhou Center for Disease Control and Prevention. The studies were conducted in accordance with the local legislation and institutional requirements. The participants provided their written informed consent to participate in this study.

## Author contributions

JX: Writing – original draft. PC: Writing – original draft. KQ: Writing – original draft. BL: Writing – original draft. ZC: Writing – original draft. ZY: Writing – original draft. CJ: Writing – original draft. YY: Writing – review & editing.
