# Supplementary material for: Association between lifestyle and dietary preference factors and conventional adenomas and serrated polyps
Source: Front Nutr. 2024 Jan 10;10:1269629. doi: 10.3389/fnut.2023.1269629 (PMC10806101; doi:10.3389/fnut.2023.1269629)
Supplement: Supplementary file 1 [file Data_Sheet_1.docx]

| **Supplementary material 1. Colorectal cancer risk assessment criteria.** | | |
| --- | --- | --- |
| **Risk Factors ^a^** |  | **Score ^b^** |
| **Age, years** | ≤ 54 | 0 |
|  | 55-64 | 1 |
|  | >65 | 2 |
| **Gender** | Female | 0 |
|  | Male | 1 |
| **Family history of CRC in first-degree relatives** | No | 0 |
|  | Yes | 1 |
| **Smoking status** | Never | 0 |
|  | Current or former | 1 |
| **BMI, kg/m^2^** | < 23 | 0 |
|  | ≥ 23 | 1 |
| ^a^ Individuals with a history of colorectal polyps or with a family history of familial adenomatous polyps in first-degree relatives were directly assessed as being at high-risk. | | |
| ^b^ A total score ≥ 5 is defined as high-risk for CRC, a total score of 1-4 is defined as middle-risk for CRC, and a total score of 0 is defined as low risk for CRC. | | |
